# Complete mitochondrial DNA sequence of the tropical hornet *Vespa affinis* (Insecta, Hymenoptera)

**DOI:** 10.1080/23802359.2017.1398622

**Published:** 2017-11-07

**Authors:** Hisashi Okuyama, Stephen J. Martin, Jun-Ichi Takahashi

**Affiliations:** a Department of Life sciences, Kyoto Sangyo University, Kyoto, Japan;; b School of Environment & Life Sciences, University of Salford, Manchester, UK

**Keywords:** Hornet, Illumina sequencing, repetitive sequence, monogynous, polygynous

## Abstract

We analyzed the complete mitochondrial genome of the Asian tropical hornet *Vespa affinis* from Ishigaki Island, Japan. It consisted of a circular molecule with 19,109 bp, which is larger to other hornet species e.g. *V. velutina*. We predicted the genome contained 13 protein-coding, 22 tRNA, and two rRNA genes, along with one A + T-rich control region. The repetitive sequences were confirmed at multiple positions in the non-coding genes. The initiation codons ATA was found in one, ATG in seven, and ATT in five genes, while the termination codons TAA and TAG were observed 11 and two genes, respectively. The average AT content of the genome was 78.4%.

There are 22 hornet (genus *Vespa*) species (Archer 2012; Perrard et al. 2013) and these have become an important model group for research into social systems such as worker altruistic behaviours, reproductive conflict, evolution of the queen number and development of sociality (Matsuura and Yamane 1990; Foster and Ratnieks 2001). The tropical hornet *Vespa affinis* is distributed throughout the subtropical and tropical regions of Asia (Archer 2012). Normally the *V. affinis* colony is founded by a single queen, although some nests contain multiple queens in tropical regions (Matsuura and Yamane 1990; Martin 1995a, 1995b), an unusual trait among the hornets (Martin et al. 2009). To date, there has been limited analysis of mitochondrial DNA from hornets (Takeuchi et al. 2017), so hampering a detailed knowledge about their population genetic structure, intraspecific phylogeny relationships, and genetic diversity. Therefore, to help resolve this issue, we analyzed the complete mitochondrial genome of the hornet *V. affinis*.

A single adult worker was collected from a colony on Ishigaki Island, Okinawa Prefecture, in the sub-tropical region of Japan (the specimen was stored in the National Museum of Nature and Science, Japan, accession number: NSMT-I-HYM 75315). Genomic DNA was isolated and sequenced using Illumina’s Next Seq 500 (Illumina Inc, San Diego, CA). The 1,462,186 reads were assembled and annotated using the MITOS web server (Bernt et al. 2013), MEGA6 (Tamura et al. 2013), and GNETYX v.10 (Genetyx Corporation, Tokyo, Japan). The phylogenetic analysis was performed using TREEFINDER (March 2011) (Jobb et al. 2004) based on the nucleotide sequences of the 13 protein-coding genes.

The *V. affinis* mitochondrial genome forms a 19,109 bp-long closed loop (accession number AP018371). Although it is ∼2000 bp longer than the *Vespa* genomes it has a very similar genomic organization, since it is composed of 13 protein-coding, 22 putative tRNA, and two rRNA genes, as well as an A + T-rich control region. The average AT content of the 13 protein-coding genes was 78.4%, similar to the 82% found in *V. velutina* (Takahashi et al. 2017). The *V. affinis* genome, was predicted to have nine protein-coding and 14 tRNA genes on the heavy strand and four protein-coding, eight tRNA, and two rRNA genes on the light strand. The genes *ND4* and *ND4L* shared seven nucleotides. Of the 13 protein-coding genes, the initiation codon ATA was found in one, ATG in seven, and ATT in five genes, while the termination codon TAA and TAG were observed in the protein-coding genes *ND4* and *Cytb*, respectively.

Phylogenetic analysis using the 13 mitochondrial protein-coding genes from 11 closely related taxa of Vespidae (Cameron et al. 2008; Chen et al. 2016; Song et al. 2016; Wei et al. 2016; Zhou et al. 2016; Fan et al. 2017; Haddad et al. 2017; Kim et al. 2017a, 2017b; Takahashi et al. 2017) was shown to be similar to the result by the combined analysis based on 45 morphological characters and six genes (Perrard et al. 2013) (Figure 1). Although more high resolution sequence data from more species may help resolve these differences. Finally, non-coding repetitive sequences in the mitochondrial DNA of *V. affinis* were confirmed at multiple positions between the 13 protein-coding genes and these regions will help to develop a suite of specific primers for the estimation of genetic relationship and genetic diversity.

**Figure 1.**
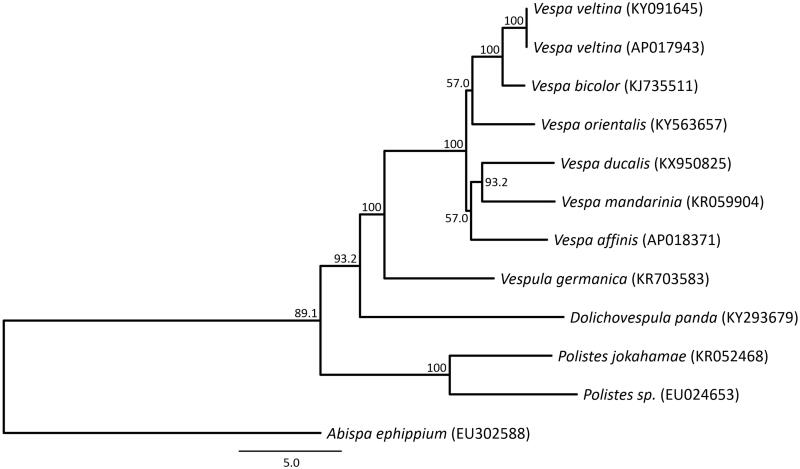
Phylogenetic relationships (maximum likelihood) of the Vespidae based on the nucleotide sequence of 13 protein-coding genes of the mitochondrial genome. The numbers at the nodes indicate bootstrap support inferred from 1000 bootstrap replicates. The sequence of Abispa ephippium was used as outgroup. Alphanumeric terms indicate the GenBank accession numbers.
